# A Vacuum-Powered Artificial Muscle Designed for Infant Rehabilitation

**DOI:** 10.3390/mi12080971

**Published:** 2021-08-16

**Authors:** Mijaíl Jaén Mendoza, Samuel Dutra Gollob, Diego Lavado, Bon Ho Brandon Koo, Segundo Cruz, Ellen T. Roche, Emir A. Vela

**Affiliations:** 1 Department of Mechanical Engineering, Universidad de Ingenieria y Tecnologia—UTEC, Lima 15063, Peru; mijail.mendoza@utec.edu.pe (M.J.M.); diego.lavado@utec.edu.pe (D.L.); 2 Department of Mechanical Engineering, Massachusetts Institute of Technology, Cambridge, MA 02139, USA; sgollob@mit.edu (S.D.G.); bkoo1104@mit.edu (B.H.B.K.); 3 Instituto Nacional de Salud del Niño de San Borja, Lima 15037, Peru; scruzb@gmail.com; 4 Institute for Medical Engineering and Science, Massachusetts Institute of Technology, Cambridge, MA 02139, USA; 5 Research Centre in Bioengineering, Universidad de Ingenieria y Tecnologia—UTEC, Lima 15063, Peru

**Keywords:** pneumatic artificial muscles, infant rehabilitation, vacuum-powered actuators

## Abstract

The majority of soft pneumatic actuators for rehabilitation exercises have been designed for adult users. Specifically, there is a paucity of soft rehabilitative devices designed for infants with upper and lower limb motor disabilities. We present a low-profile vacuum-powered artificial muscle (LP-VPAM) with dimensions suitable for infants. The actuator produced a maximum force of 26 N at vacuum pressures of −40 kPa. When implemented in an experimental model of an infant leg in an antagonistic-agonist configuration to measure resultant knee flexion, the actuator generated knee flexion angles of 43° and 61° in the prone and side-lying position, respectively.

## 1. Introduction

Almost 14% of the population in the US suffers from a motor disability such as quadriplegia, which prevents them from performing activities of daily living [1] and leaves them dependent on family members or assistive devices such as wheelchairs. There are multiple reports of assistive devices for adults affected by motor disability in the upper and lower limbs [2,3,4], but there has been less focus on devices intended for children and infants with motor disabilities. Specifically, for lower limb rehabilitation in infants less than a year old, there is a lack of devices designed specifically for stimulating leg movements, which is important for early motor development [5,6].

Continuous exploration and trial and error experiences are important contributors to the acquisition of gross motor function [7]. For example, infants typically explore early movement such as spontaneous kicking, stepping, and crawling before developing the ability to walk. Previous work concludes that mature locomotor movement is dependent on preceding alternating movement such as flexion and extension of the lower limb joints [8,9]. From these findings, it can be concluded that passively training and preserving early movements such as kicking and stepping in infants with motor dysfunction can be beneficial for the development of typical locomotor skills in later life. Existing work has supported this logic in the development of wearable lower-leg active devices for infants [10].

One condition that can benefit from the strategy of infant passive leg rehabilitation is myelomeningocele (MMC), a neural tube defect involving incomplete development of the spinal canal, leading to a protruding sac containing the spinal cord, cerebrospinal fluid, and meninges. Due to neural damage, infants with MMC suffer loss of motor function in the lower limbs. As a consequence, in their adulthood, they are dependent on assistive devices including orthoses, wheelchairs, crutches, and walkers to mobilize [11]. In the USA, the incidence of MMC is approximately 1500 to 2000 infants per year [12]. Treatment of infants with MMC involves a multidisciplinary approach and attempts to enable patients to reach the maximum level of development possible, given the presence of a neurological lesion. It includes passive mobilization of lower limbs, use of devices, and increased contact between parent and child. The objective is to stimulate the development of healthy muscles to decrease the likelihood of paralysis or paresis [13]. Rehabilitative treatment begins at birth and continues throughout development.

Physical therapy consists of gentle passive exercises on the infant’s main joints and includes stretches to improve the range of motion of the joint and muscle strength [14]. The application of such a device can be extended to infants with motor developmental delays and other motor disabilities such as cerebral palsy (CP). Wearable and autonomous robotic devices can assist with rehabilitation exercises and lower the associated cost and barrier to rehabilitation, especially for families living in areas without easy access to health professionals and physiotherapists. Currently available rehabilitative technology often employs traditional rigid robotic exoskeletons [15,16,17,18,19] to supplement the motion of the wearer. Commercially available state-of-the-art pediatric exoskeletons, like the ATLAS 2020 [20], can provide high support torques and large degrees of freedom but use rigid parts and are heavy and bulky. Additionally, the ATLAS 2020 is only available for children from 3–14 years of age.

To overcome these limitations with rigid exoskeletons, previous research has focused on soft robotic exoskeletons and assistive suits, though there is still a paucity of research on soft suits for infants. Soft robotics has potential in this context due to its lightweight, low-cost, and conformable nature that can safely interface with humans. One of the earliest soft actuators, the McKibben actuator, was designed for use in an active exoskeleton for assisting the motion of paralyzed hands [21]. In the current literature, utilization of soft robotic exoskeletons include gait assistance via ankle [3] and hip [2] support, as well as hand motion support and rehabilitation via pneumatic actuators designed to match finger motions [22,23]. For children above the age of three, active suits have been proposed that are powered by soft actuators at the shoulder [24] and both shoulder and elbow [25]. An active ankle exoskeleton powered by pneumatic actuators has also been proposed for pediatric use [10], and previous work has developed cable-driven robots to provide external forces to children with CP as a method to improve gait [26]. There is limited work on soft exoskeletons for infants in the range of 0–12 months of age, but proposed work includes a soft passive upper-limb support suit [27], as well as an active sleeve for aiding in exploratory leg motions [10].

A dominant actuation scheme in soft robotics is that of pneumatic actuators, which use physical constraints around a soft pressurized bladder to perform complex motions [28]. This type of actuator has been thoroughly studied and has been shown to produce a variety of motions, such as linear contraction [21,23,29,30,31], bending [23,32,33], torsion [23,31,34], or complex multi-axis motion [23,31,35,36], making it an attractive option for the complex motions required for rehabilitative suits.

Though the aforementioned soft exoskeleton work, and most of the work in the field uses primarily positive pressure pneumatic actuators [5,6,28,37,38,39], interest in vacuum powered artificial muscles (VPAMs) has increased in recent years. Beyond being able to perform the same variety of motions as positive pressure actuators [40,41,42,43,44], often at larger contraction ratios [41,45], VPAMs are well-suited for wearable applications, as they can be actuated in space-limited scenarios and may be safer than actuators driven by positive pressure, as the negative pressure would be less likely to injure a wearer in case of rupture. The space-limited capability is particularly accentuated in the case of a wearable device for infants, given their small size as an end-user group.

One example of VPAMs, referred to in previous work as bellows actuators, consist of an inextensible polymeric skin over a series of rings that contract linearly and produce large forces with high contraction ratios (≈90%) [41,45]. Despite these benefits, these actuators experience hysteresis and can require positive pressure to return to their original state. Yet another group of VPAMs is the Fluid-driven Origami Artificial Muscle (FOAM) in which a zigzag skeleton structure is encased in a polymeric skin to produce large contractions, from 50% to 90%, and operating pressures from 0–70 kPa [46]. This actuator can be fabricated at small scales (7.3 mm × 20 mm × 60 mm), but its contraction and force are hindered by unpredictable buckling or sliding of the skeleton inside the skin. Another skeleton-skin system following a similar concept uses a magazine spring (a compression spring of rectangular coil shape) to achieve a linear contraction ratio of 50% at a similar operating pressure to the FOAM, while keeping a low cross-sectional profile [47]. One major drawback for this actuator is the relatively complex fabrication method of the spring [47].

Given the clinical need for, and lack of existing work on, active lower-limb rehabilitative exoskeletons for infants, and given the unexplored benefits of VPAMs in wearable technologies, we propose a low-profile vacuum-powered artificial muscle (LP-VPAM) for use in the rehabilitation of infants of six months or younger. The actuator was inspired by the bellows, FOAM, and magazine spring concepts: it consists of a low-profile FOAM-like skeleton oriented in a manner similar to the magazine spring with a gap between the skeleton and surrounding skin so that it mimics a bellows actuator early in its contraction. The design can be miniaturized (10 mm height profile) and operates at low vacuum pressure magnitudes (<40 kPa) for increased safety. The zigzag skeleton oriented correctly on the leg leverages the ease of fabrication of the FOAMs-style skeleton while allowing for a large contraction despite the low-profile form, which would not be possible with a bellows actuator, as is discussed in a later section. Additionally, we address the issues of slippage and buckling of the skeleton associated with FOAMs by attaching the skin to the end rectangular fittings of the skeleton. We demonstrate the actuator’s application through the knee angle output in two specific stretches for babies on an infant leg model, where the actuator drives knee flexion. In the first leg rehabilitation exercise, the leg model is in simulated the prone position, and in the second, the leg model is in the simulated side-lying position.

## 2. Materials and Methods

### 2.1. Vacuum Actuator Design

As explained above, our actuator was inspired by the Fluid-driven Origami Artificial Muscle (FOAM) [46], magazine spring [47], and the bellows [41] concepts. In order to adapt the actuator for our application, we considered the following criteria: size, operating pressure, and range of motion. In rehabilitation exercises in infants, the volume of the actuator is limited by the anthropometric measurements of the infants such as circumference and length of the lower limbs. Thus, a low-profile actuator is suitable to avoid contact and interference with the natural movements of the wearer, while the cross-sectional area (CSA) should be large enough to generate sufficient force output. Furthermore, the operating pressure of the actuator should be minimized in order to reduce risk of injury. Finally, high levels of contraction are necessary to achieve the desired range of motion in the rehabilitation exercises [41,46]. The range of motion of the knee angle of healthy infants for a kicking motion is approximately 54 degrees [48], and this angle range was chosen as the benchmark value for our actuator to reach when applied to a model infant leg. In the physical therapy, the knee angle is the main output to validate the application of the VPAM.

**Figure 1:**
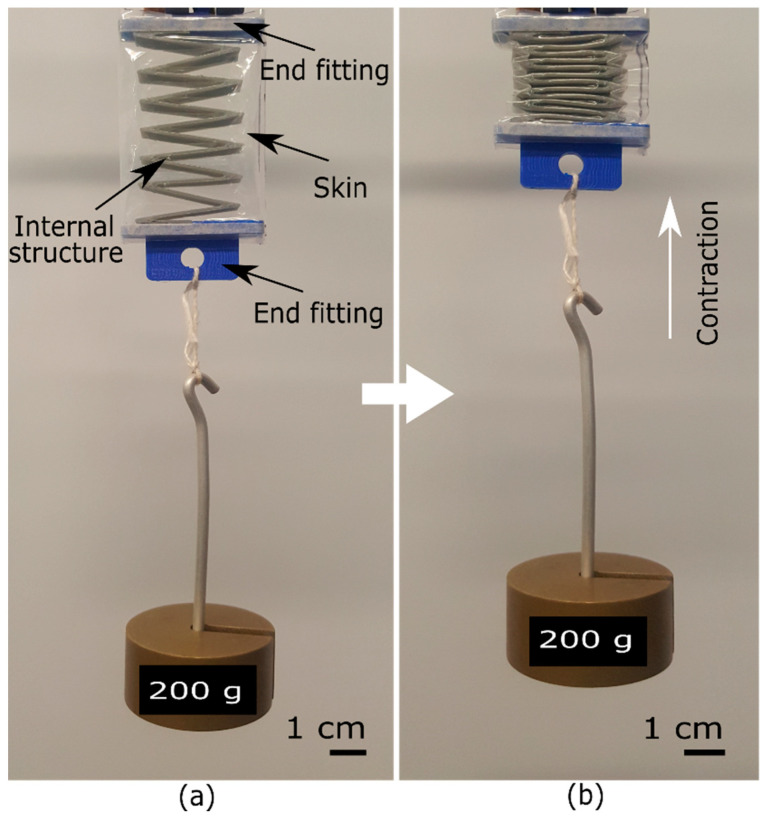
Our low-profile vacuum powered artificial muscle LP-VPAM with a load of 0.2 kg directly powered by an electric vacuum pump (∆ = −40 kPa). Unactuated state (**a**) and actuated state (**b**).

The LP-VPAM is composed of three components: a zigzag structure or skeleton, end fittings, and a skin. Though our actuator employs similar components to the FOAM concept, we have attached the structure to the skin using end fittings to avoid sliding of the structure inside the skin, which would diminish the output range of motion in rehabilitation exercises. The relaxed and pressurized states of the actuator are shown in Figure 1a,b respectively.

**Figure 2:**
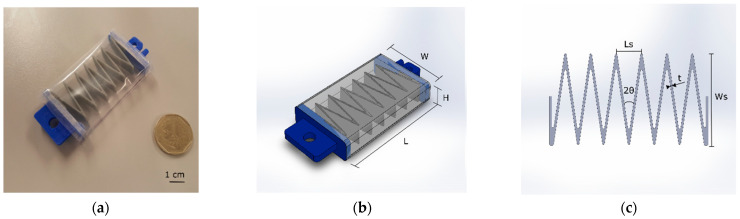
Image of assembled actuator with reference scale (**a**) and labels of geometrical parameters of actuator (**b**) and zigzag skeleton (**c**).

The zigzag skeleton is a folding structure composed of V-shaped cells. A cell has one compliant hinge that enables folding and acts as a compression spring. In contrast to other artificial muscles that use 3D structures and vacuum pressure, a zigzag structure enables flat actuators with a rectangular CSA, instead of employing a circular CSA and a tubular skin that produces less force when its diameter is minimized to the same profile (or height H as shown in Figure 2) as the LP-VPAM [41,45,49]. The thickness of the zigzag structure (t as shown in Figure 2) was set to 0.8 mm with a maximum overall length (L) of 60 mm, based on of the performance advantage of thinner walls and longer structures reported for FOAM actuators [46]. Furthermore, the angle of the V-shape cell (2θ) was 18°.

In our experiments, we chose four geometrical variations for this actuator. Firstly, we established from the 54° desired angle range that a minimum contraction of 30 mm was necessary. This was extrapolated into a minimum number of four cells for an actuator. We then chose a six-cell option to provide a 50% increase in the theoretical contraction beyond the required amount, expecting that our geometrical estimates would over-estimate the actuator’s true contraction. Finally, to assess the trade-off between lower height (better form-fitting) and lower output force (higher actuation pressure), we selected two values for the skeleton height (H) of 5 and 10 mm. The four permutations of these dimensions and the given actuator configurations are included in Table 1 below, where Ws is the skeleton width, H is the skeleton height, Ls is the skeleton cell length, W is the actuator width, and C is the number of cells.

### 2.2. Fabrication

The fabrication process consisted of three steps: (1) 3D printing the internal structure, (2) fabricating the encapsulating skin, and (3) assembling the vacuum actuator. For rapid, facile prototyping, we employed polyethylene film (PE, Policlick, Lima, Peru) for the skin and built the structure with Filaflex (TPU filaments) and the end fittings with Polylactic Acid (PLA, eSUN Industrial Co., Shenzhen, China) printing filaments using a FlashForge Creator Pro 3D Printer. The end fittings are rectangular, rigid components that allow the skeleton and skin to be hermetically attached to each end of the actuator with one end connected to a pneumatic tube. In the second step, we marked the PE film (0.1 mm thickness) to align the end fittings in the skin. Next, we put masking tape on the markings on the PE film where the end fittings are subsequently attached using a fast-drying cyanoacrylate adhesive to create an airtight seal. The masking tape acts as an intermediate material between the end fittings and the skin, as the adhesive cannot directly adhere to the PE. Finally, before the assembly of the actuator, the internal structure was attached to the end fittings. The actuator was assembled by gluing each side of the end fittings to the tape in the skin to avoid sliding of the structure inside the encapsulating skin during actuation. At the end of the assembly, one longitudinal edge of the actuator was heat sealed to form the encapsulating skin. A heat sealer (HD-300M-IR, China) was used with a sealing time of 4 s working at 300 Watts. The assembled actuator and the geometrical parameters of the actuator and zigzag skeleton are shown in Figure 2a–c, respectively, where W is the width of the actuator, H is the height of the actuator, L is the length of the actuator (distance between end fittings), W_s_ is the skeleton width, L_s_ is the length of one actuator cell, 2θ is the angle of the V-shape cell, and t is the thickness of the skeleton.

### 2.3. Finite Element Modeling of the Structure

**Figure 3:**
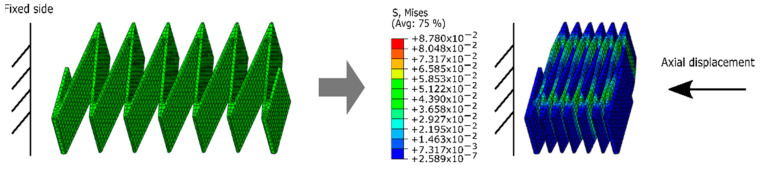
Schematic of the Finite Element Model setup of the skeleton, displaying stress concentrations for the uncontracted (constant zero-stress) and fully contracted states.

The actuator’s contractile force is defined by the vacuum pressure and resisted by the skeleton’s restoring force. Before fabricating the chosen skeleton geometries, we wanted to ensure it would have a low restoring force (i.e., low stiffness) relative to the actuator’s output forces, allowing for lower operating pressures for the actuator. Towards the end of predicting the skeleton’s low stiffness, we created a Finite Element Model (FEM) of the skeleton structure on the software ABAQUS/CAE (Simulia, Dassault Systemes, Providence, RI, USA). The model consisted of applying an axial displacement to one end of the structure, while fixing the other end as is shown in Figure 3. We assumed the structure displacement to be a pure linear contraction, thus, the translation and rotation in other axes were constrained at both ends. A general contact boundary condition was applied to the model with hard normal contact and frictionless tangential contact (as normal reaction forces dominate this system). Though the FilaFlex is hyper-elastic, the low expected strains (below 0.15) led us to assume a linear elastic material model with a Young’s modulus of 38.9 MPa. This stiffness value was extracted from tensile tests (Instron 5944) of 3D-printed strips, as described in the Supplementary Materials. The maximum strain in the FEM was later confirmed to be below 0.05, confirming the validity of our linear elastic assumption. The skeleton structure was modeled using linear hexahedral solid elements (C3D8R).

### 2.4. Modelling Actuator Force Output

As the design section above describes, this actuator is centrally based on the FOAM design [46], but because the width of the skeleton is smaller than that of the actuator as a whole, the actuator operates in two phases: before the skin contacts the skeleton, it operates as a bellows actuator [41], and once the skin begins to contact the skeleton, it transitions into behaving like a FOAM actuator.

To simulate these two phases, and better predict the actuator’s force output and contraction numerically, we developed a model of the actuator using the virtual work principle, following the process previously described [50]. Briefly, the actuator is approximated as a 2D geometry and the volume loss over its contraction is geometrically extracted in a numerical algorithm. The derivative of the volume loss throughout contraction is applied to Equation (1) [50]:

(1) $$F=A_{c}\times P\times \left(1-\frac{dV_{r}}{dh}\times \frac{1}{D}\right)$$

where $A_{c}\;$ is the actuator’s cross-sectional area, P is its actuating pressure, $V_{r}$ is the actuator’s volume loss, h is the variable symbolizing contraction, and *D* is the characteristic diameter, in this case, the width of the actuator (30 mm). This equation calculates the actuator’s quasi-static force output for all given points through its contraction, called a force-contraction profile (FCP).

**Figure 4:**
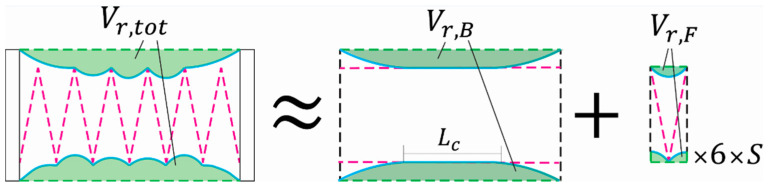
Schematic of the two geometrically-based models for approximating the volume loss of the actuator throughout its contraction, showing the bellows and FOAM models labeled with the *B* and *F* subscripts, respectively. The volume loss from the FOAM model is multiplied by six and a scaling factor proportional to $L_{c}$.

Because of this actuator’s compound behavior, its volume loss curve was created by the super-position of two volume curves. First, a model of a bellows actuator with a width of 56 mm and a contact boundary 5.16 mm below the skin is built. This represents the bellows behavior before the actuator contacts the top of the FOAM skeleton. Second, a model of a FOAM with a characteristic diameter of 27.7 mm and a cell width of 9.3 mm is built (the model assumes a zero-thickness skeleton). These two parallel models are shown in Figure 4. The value of 9.3 mm comes from the total length of the actuator (56 mm) divided by the number of zigzags in the skeleton (six). The volume loss from the FOAM model is then multiplied by six to match the six cells and added to the volume loss from the bellows actuator following Equation (2):

(2) $$V_{r,tot}\left(h\right)=V_{r,B}\left(h\right)+S\left(h\right)\times 6\times V_{r,F}\left(\frac{h}{6}\right)$$

where h is the variable representing the current point in the actuator’s contraction (the actuator’s current length), and S(x) is a scaling factor calculated by dividing the current length of skin contacting the boundary in the bellows model ($L_{c}$ in Figure 4) by the total possible skin contact length for the bellows model (56 − 5.16 × 2 = 45.7 mm). The B and F subscript apply to the separate bellows and FOAM models (Figure 4). This scaling factor allows for a smooth superposition of the FOAM model volume loss onto the bellows model to assemble an approximate function of the actuator’s volume loss throughout its contraction. The super-imposed volume loss is then used in Equation (1) above to generate an FCP for the actuator.

Finally, once the model is calculated, a shifting and scaling factor are applied to capture the system losses that are not included in the idealized model. The shift used was 2% and the scaling factor followed the equation previously described [50]. Using a Young’s modulus of 110 MPa and thickness of 0.1 mm for the skin (manufacturer values), the magnitude scaling factor was 0.48.

.

### 2.5. Actuator Restoring Force Experiment

The virtual-work model of the actuator is idealized and does not include losses, such as the nonlinear restoring force that the actuator produces as the skeleton and skin are compressed. To supplement the idealized model, we performed a compression test on the actuator without applying a vacuum to extract this restoring force. Though the FEA described above approximates the restoring force, the presence of the skin and the nonlinear effect at the end of the contraction necessitate experimental measurements.

The actuator was held on both ends with a 2 kN load cell on the mechanical tester, a pre-tension of 1 mm was applied, and the actuator was compressed at 60 mm/min without a vacuum. Importantly, the skin configuration in between the spaces of the skeleton’s zigzag towards the end of the contraction (Figure S2) needs to be captured, as this is an important contributor to the restoring force. To achieve this, a second set of experiments was performed, where the −40 kPa actuation pressure was applied until 20 mm of contraction was achieved, after which the actuator was subjected to atmospheric pressure. The force output for the remainder of the contraction better captures the final resistance values for the actuator, and these force curves were appended to the measurements from the first set of experiments. The experiments were performed on two actuators (*n* = 2) with two replicate experiments each.

### 2.6. Quasi-Static Characterization of the Actuator

To evaluate the virtual-work-based model of the actuator, the actuator’s FCP is measured using a mechanical tensile tester (Instron 5944). First, the actuator is held at both ends at its full uncontracted length using a 2 kN load cell (Figure S1) and placed under 1 mm of pre-tension, then −40 kPa is applied to the actuator using a manual vacuum pressure regulator (IRV10-N07). The actuator is then allowed to contract at a rate of 60 mm/min for a total displacement of 35 mm from the initial uncontracted length (after which point the skeleton is fully collapsed) and the force output is extracted throughout the contraction. This experiment used two actuators (*n* = 2) with two trials each.

To characterize the fixed force output of the actuator, we built a setup to measure the contraction force composed of a force sensor (DFS-BTA, Vernier, OR, USA), a Lab Quest Mini (LQ-MINI, Vernier, OR, USA), and a universal support that acts as a rigid frame. The end fitting in the top of the actuator includes a linkage that enables the actuator to connect to the force sensor, while the bottom of the actuator is attached to a metallic tweezer to hold it fixed at full length. To conduct the test, the vacuum pressure was applied as a ramp from 0 to −40 kPa and it was repeated three times. This experimental setup can be seen in Figure S3.

Furthermore, a different configuration was used to measure the maximum absolute contraction of the actuators subjected to a load (Figure S4). The actuator was restrained in a horizontal position using a 3D printed piece and connected to a pulley through an inextensible Kevlar cable to which a load of 0.5 kg was mounted.

Additionally, the same configuration was employed in order to obtain the maximum contraction produced by the actuator by varying the load. We tested different loads from 0.1 kg to 0.5 kg in 0.05 kg increments. The maximum contraction was registered when the applied vacuum pressure was −40 kPa. The experiment was repeated three times and the data were extracted from videos in Tracker (Open Source Physics, Available online: https://physlets.org/tracker/ (accessed on 10 May 2021)). This setup allowed us to compare the stiffness of the actuators.

### 2.7. Dynamic Response of the Actuator

The actuator was clamped at one end such that it was oriented horizontally, and the unconstrained end was attached to a 200 g mass through a string that passed through a pulley that allowed the mass to hang freely, applying a constant tensile force to the actuator. A step input of −20 kPa in pressure was applied to that actuator, and videos of the step response were captured and processed in Tracker software to quantify the displacement of the actuator’s free end. The components used for this test were a vacuum pump (AIRPO D2028B) and a vacuum pressure regulator (ITV 0090) connected to an Arduino Mega. The experiment was performed on four actuators (*n* = 4) and repeated three times per actuator.

### 2.8. Case Application in Rehabilitation Exercise

To demonstrate the capabilities of the actuator, a setup including a 3D-printed leg model of a 6-month-old infant was built in order to carry out flexion and extension movements of the knee joint, allowing pure flexion of the leg model. The purpose of the setup was to measure the output knee angle of the leg model and evaluate whether the output angle reached the desired output range (54 degrees). The anthropometric measurements for a six month old infant were taken into account for the model leg dimensions and the weight of the lower limbs were also incorporated [51,52]. At the ankle position, a 0.1 kg mass was added representing the foot weight. The actuators are attached to the model leg in the anterior and the posterior side of the thigh, through two anchor points per actuator. One proximal anchor point is close to the hip, while the distal anchor point is close to the ankle. The anchor points positions were chosen in order to reduce the required force for the exercises. This experimental setup can be seen in Figure S5.

We tested the antagonist-agonist configuration of the actuators in two positions inspired by the rehabilitation exercises related to the knee joint. In both the prone and side-lying positions, we evaluated the angle produced by the antagonist-agonist configuration in cycles of knee extension and flexion. For the rehabilitation exercises, VPAM with six cells and a height of 10 mm was used for extension motion and the same actuator with nine cells was employed for the flexion motion. A large actuator was chosen for the second motion because in order to produce a high knee flexion angle for that motion, the contraction had to be larger. The actuator was controlled by the vacuum pressure regulator at −40 kPa and the experiment was repeated ten times.

## 3. Results

### 3.1. Finite Element Modeling Results

**Figure 5:**
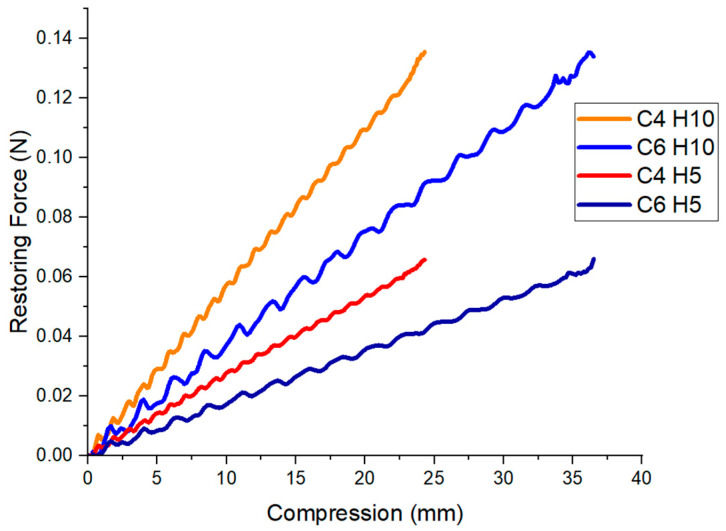
Force over contractile displacement extracted from the skeleton Finite Element Modelling of the four and six-cell skeletons with width of 30 mm as previously described, where C stands for number of cells and H stands for skeleton height. A skeleton cell is defined as one V-shaped section in the zigzag pattern of the skeleton. Skeletons were compressed to the point of self-contact at the end of their compression.

Figure 5 shows the relationship between the compression and the restoring force obtained in the FEM. The results of all the skeleton configurations are ordered by number of cells of the structure (C) and skeleton height (H). As shown, the restoring force increases proportionally with the compression in our four permutations. When the compression achieves the maximum value set in the FEM model (e.g., 25 mm for C4 and 35 mm for C6), the FEM values for peak force magnitudes are below 0.15 N in our four skeletons. Details of the stiffness of the skeleton material are included in the Supplementary Materials (Figure S6).

### 3.2. Actuator Force-Contraction Profile

**Figure 6:**
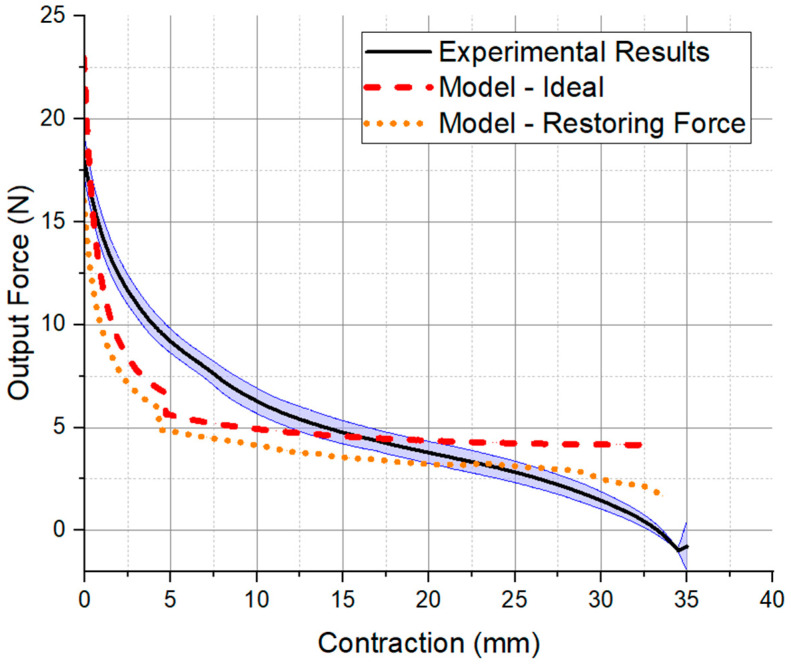
Force-contraction profile of actuator from the average of experimentally measured results (*n* = 2 actuators, two trials each) compared with the virtual-work based model after a scaling factor of 0.48 and discarding the first 2% of the model’s contraction. The ideal model is as presented in Equation (1), while the restoring force curve is the result of subtracting the experimental average of the restoring force of the actuator (see Supplementary Materials) from the ideal model.

Figure 6 shows the results for the quasi-static FCP experiments performed on the actuators averaged over all four trials. The data show that the trials were highly repeatable as the standard deviation is approximately 0.4 N. The experiment is compared with the virtual work model in its ideal form and after the subtraction of the restoring force from the actuator restoring force experiments (averaged over all four trials). The maximum force value is approximately at the beginning of the contraction, then, it gradually decreases. As shown, the experimental force follows a similar behavior to the ideal model; however, the force output decreases much faster after 15 mm of contraction.

### 3.3. Quasi-Static Characterization of the Actuator

**Figure 7:**
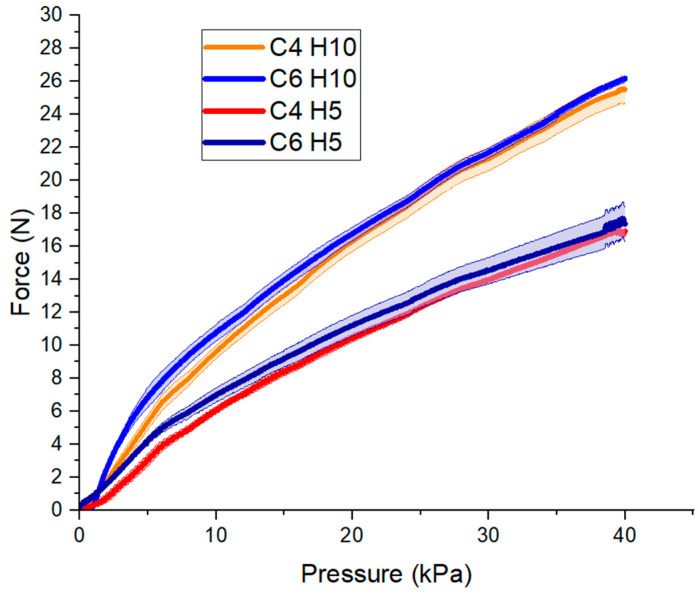
Force versus actuation vacuum pressure magnitudes for actuators held fixed at uncontracted length, where C stands for number of cells and H stands for skeleton height (in millimeters).

The results of the fixed force output characterization are plotted in Figure 7; H is the skeleton height and C is the number of cells. Note that there are small differences between the actuators that have the same H, which might be produced by differences in fabrication, and there is a break force gap between the actuators with H5 and H10, despite having the same C. As the actuators with the same height have the same cross-sectional area, the output force is the same.

**Figure 8:**
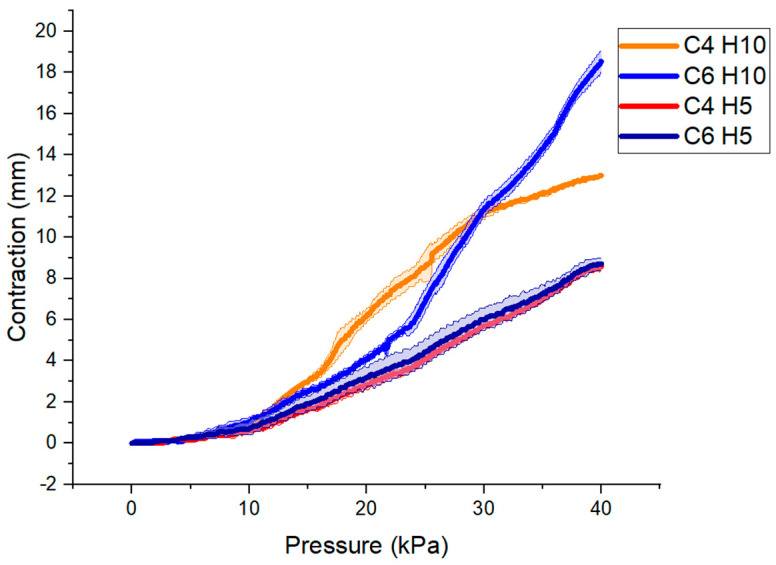
Maximum contraction state versus actuation pressure for actuators with four and six cells (C4 and C6) and heights of 5 and 10 mm (H5 and H10), supporting a 500 g mass. Maximum contraction defined as total displacement from the initial actuator length before reaching equilibrium due to the applied load.

From the second setup in the characterization, the contraction–pressure curve is plotted in Figure 8, where the maximum contraction was approximately 19 mm for the largest actuator (C6 H10). The results show that the low-profile vacuum-powered artificial muscle LP-VPAM with C6 H10 might be suitable for the rehabilitation exercises in which the load is less than 0.5 kg (Figure 8).

**Figure 9:**
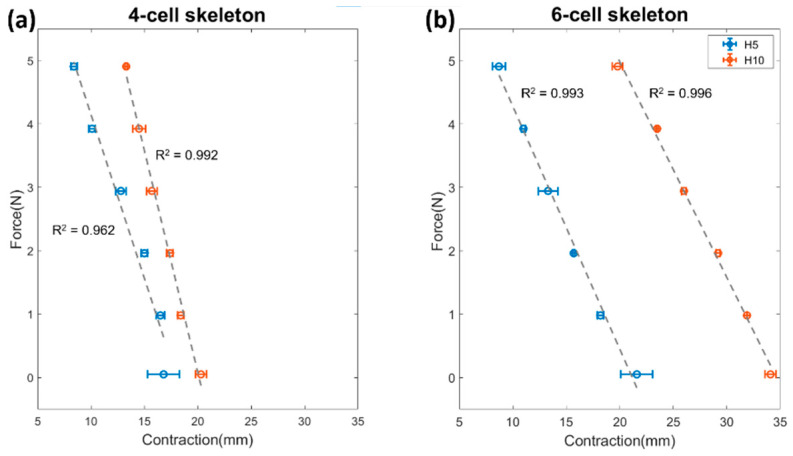
Force–displacement curve for actuators with varying number of cells and skeleton heights (H) given an actuating pressure of −40 kPa. The curves for actuators with 4 cells are shown in (**a**) and those with 6 cells in (**b**). The linear fit slopes of these curves were used to estimate a linear stiffness for each actuator using Hooke’s law.

In Figure 9, it can be seen that the force–contraction relationship is linear for all the actuators. The actuators with the larger cross-sectional area (H10 mm) produce the larger contraction for each load (Figure 9a,b). Given that the contraction is inversely related to the axial displacement of each actuator, the negative of the slope for each actuator’s linear fit line is the stiffness for that actuator. Those values are shown in Table 2.

### 3.4. Dynamic Response of the Actuator

**Figure 10:**
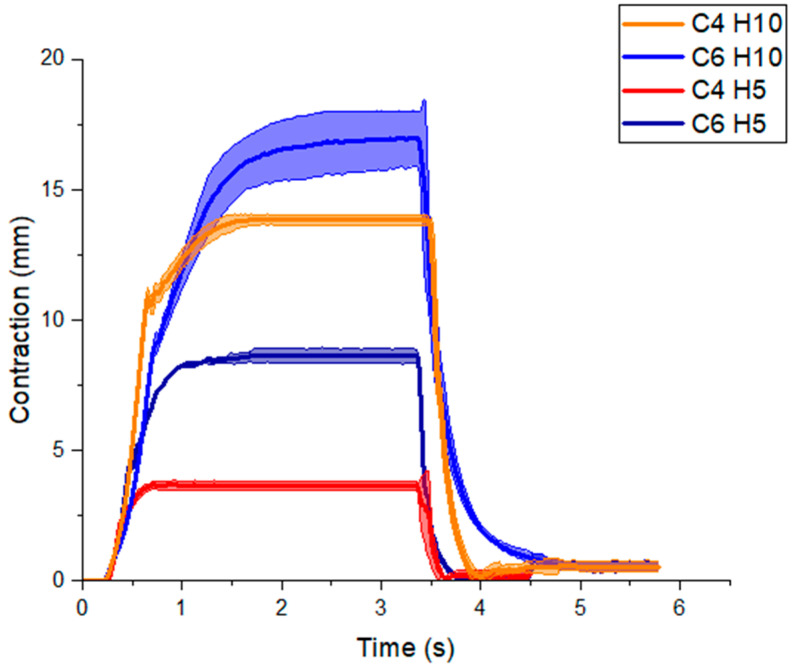
Dynamic response of actuator contraction. Displacement of unconstrained end of actuators with 4 and 6 cells (C4 and C6) and heights of 5 and 10 mm (H5 and H10) over time, given a step input of −20 kPa vacuum pressure. A 200 g mass hanging from a string passing through a pulley is attached to the unconstrained end of the actuator, which is oriented horizontally.

Figure 10 shows the dynamic response of the four actuators attached to the 200 g mass after a step input of −20 kPa. The results of VPAMs with four-cell skeletons (C4) and with six-cell skeletons (C6) are shown in Figure 10.

### 3.5. Case Application in Rehabilitation Exercise

**Figure 11:**
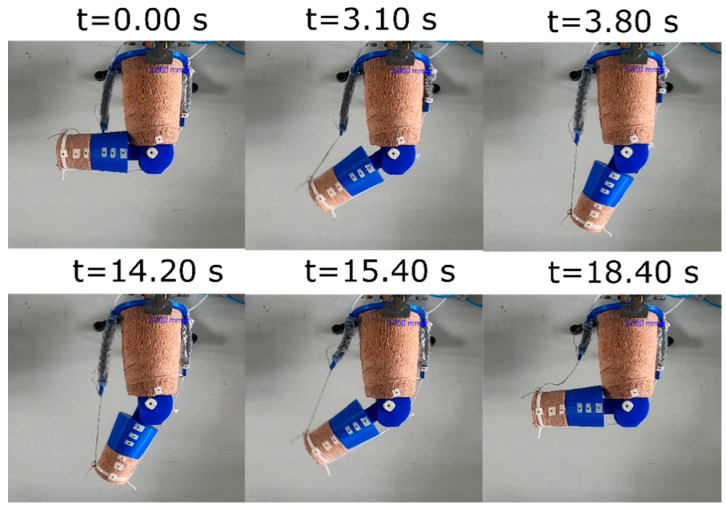
Time series images of infant leg model to exemplify rehabilitation exercise under extension (top three frames) and flexion (bottom three frames) as actuators are respectively contracted. Leg is fixed sideways to represent infant lying on their side, the direction of gravity being into the page.

From the experimental results in the characterization and in the dynamic response, we hypothesize the actuator (C6 H10) would be suitable to lift the leg or flex the knee joint for rehabilitation exercises with infants in an agonist–antagonistic configuration. The benefit of this configuration is that the agonist muscle actuator produces the extension motion and it does not employ the antagonistic muscle actuator to return to its relaxed state. Returning the infant’s leg to the resting position would require a significant output force of the antagonistic muscle actuator. An example of the muscle configuration working on the leg model is shown in Figure 11, where the agonist muscle produced knee extension and the antagonistic muscle caused knee flexion.

**Figure 12:**
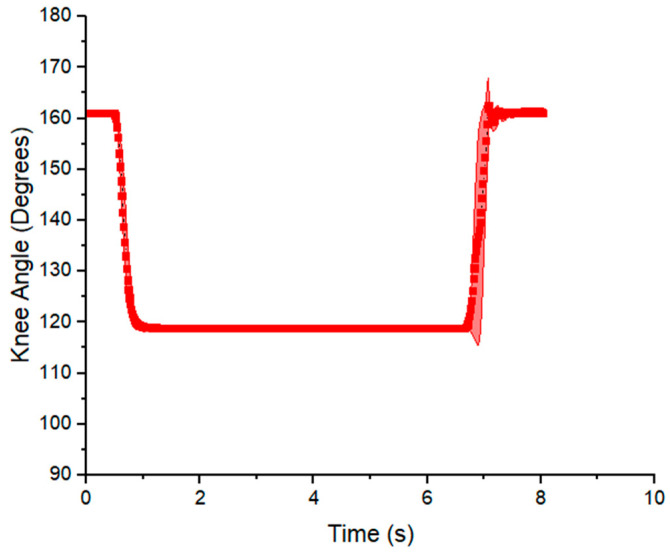
Knee flexion angle over time from model leg experiment in the prone position, using the C9 H10 actuator. The angle is measured between the thigh and lower leg, such that 90° would represent the knee at a right angle and 180° represents the leg fully extended such that it is in a straight configuration. The value shown is the mean with standard deviation for *n* = 10 cycles.

**Figure 13:**
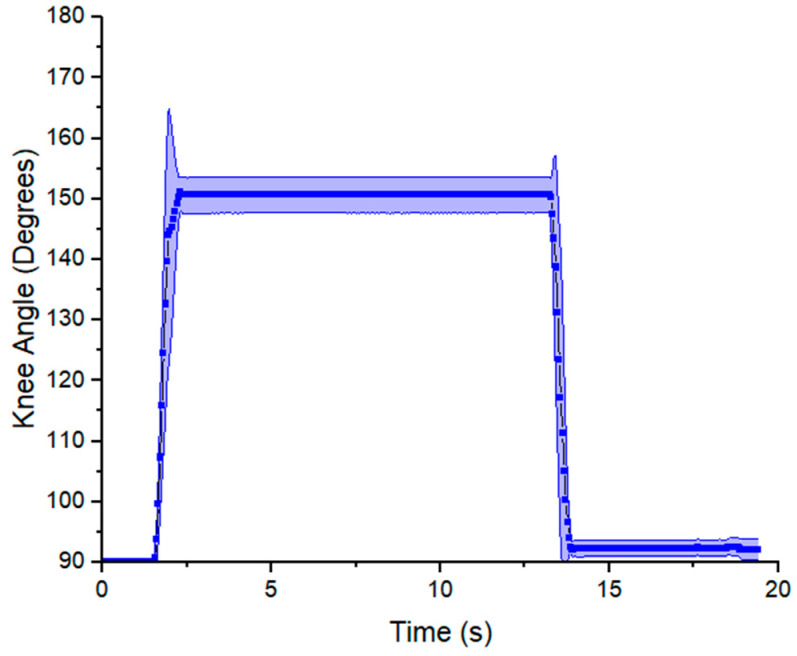
Knee flexion angle over time from model leg experiment in the side-lying position using the C6 H10 and C9 H10 actuators. The same angular nomenclature is used from the previous figure. The value shown is the mean with standard deviation for *n* = 10 cycles.

The angle–time curve responses are shown in Figure 12 and Figure 13 for prone and side lying position, respectively. The angle is measured between the thigh and the lower leg. At 90 degrees the knee is at a right angle and at 180 degrees the leg is fully extended. The angle was approximately 119 degrees in the prone position for flexion. As shown, the angle decreases abruptly by flexion and then it returns to its initial value by extension (160 degrees). The data show that the experiment was highly repeatable for each cycle of exercise (number of repetitions = 10), the standard deviation (SD) was two degrees in the flexion motion and six degrees in the extension motion. Figure 13 shows a similar behavior in the side lying position; however, the cycle of exercise starts with an extension motion and finishes with a flexion motion. The measured angle increased abruptly, achieving a maximum value of 151 degrees by extension and then decreased to 92° by flexion.

## 4. Discussion

### 4.1. FEM of Different Skeleton Parameters

For all designs, the restoring force remains below the target threshold of 0.15 N. As expected, the increased height of a skeleton (5 to 10 mm) causes an increase in stiffness and thus higher restoring force resisting contraction. This will be relevant in the following sections, as the taller height skeleton allows for a larger actuator cross-section and thus larger output force, trading off with a larger restoring force. Increasing the number of cells decreases the per-unit-distance stiffness of the actuator proportional to the ratio of the number of cells, as expected given the dimension of a given cell does not change. An assessment of the accuracy of these FEM results compared to experimental results is included in the Supplementary Materials (Figure S7). Briefly, we find that the FEM under-estimates the stiffness by half, possibly due to a combination of increased thickness after printing, under-estimated material stiffness from testing, and anisotropy of 3D printed skeleton fiber orientation.

### 4.2. Actuator Force-Contraction Profile

As Figure 6 shows, the force magnitudes and decreasing force trend agree with the output of the actuator. The deviation in the shape of the FCP is improved somewhat with the addition of the experimentally measured actuator returning force, as it captured the nonlinear increasing restoring force that creates the characteristic drop in output force at the end of the actuator’s contraction. However, the deviation in shape remained, which may be in part due to the superposition approach to the model, as opposed to a single geometrical approximation of the entire actuator (a functionality that is not included in the current program, but which could be implemented in the future).

Considering the idealized nature of the model and rapid solution time, the information contained in the estimated FCP is sufficient to help in the estimation of the system’s initial output force (an error of only −15%), general force magnitudes, and approximate maximum contraction.

One important point that this model demonstrates is the value of the skeleton in this low-profile actuator design. If the actuator was built like a bellows actuator (no skeleton), the short cross-sectional dimension of the actuator would dominate, and it would have the output of a bellows actuator with a characteristic diameter of 10 mm and ring spacing of 56 mm, meaning its total contraction would be limited to a maximum of 10 mm with a highly variable force output (high at the beginning and low at the end of the contraction) [41,50].

### 4.3. Characterization of the Actuator

In Figure 7, the force-pressure curves approximate a linear trend. As expected, the maximum break force output registered was 26.4 N for the largest actuators. This behavior is dominated by the CSA of the actuator, which is proportional to the output force according to Equation (1). Additionally, increasing the number of cells (C) of the actuator had a minor effect on the force output. LP-VPAM can be employed for rehabilitative treatment in babies, since it can produce low force above the baby’s leg weight. The force output can be higher than the required value, however it can be controlled as it is proposed in [50].

In comparison to other vacuum actuators in Table 3, the scaled force output from our actuator is higher than FOAM actuators with a magazine spring [47]. Our design has a low stiffness structure, translating to a lower operating pressure and a higher scaled value. Scaled output force from FOAM actuator is slightly higher due to differences in materials selected for the skin. Since the Bellow actuator is composed of rings without an encapsulating skeleton, its scaled output force is higher. The LP-VPAM design can be designed to produce higher force magnitudes. Furthermore, the C6 H10 VPAM achieved a maximum contraction of 61%, which is comparable to other VPAMs.

In Figure 8, the actuators demonstrate similar contraction for low vacuum pressures, and diverge at higher vacuum pressures. This phenomenon might be explained by the interaction between the skin and the skeleton. When the skin contacts the skeleton, it is more difficult for the air to freely travel through the actuator. Similarly, a bellow type vacuum pneumatic actuator has shown that self-intersection of the skin can limit its stroke [39]. Furthermore, in some cases such as the C6 H5 and C4 H5 actuators, the performance of the LP-VPAM is reduced due to out of plane bending. Non-linearities are observed in Figure 9 due to the out-of-plane deformation with small (<0.1 kg) or large (>0.5 kg) masses. For the range of forces tested, there is no clear trend in the stiffness between actuators with the same number of cells, which may be due to a combination of factors such as the globally nonlinear FCP of these actuators, as well as variations in fabrication. However, the estimate that an increased number of cells leads to a decreased stiffness from the FEA of the skeleton holds true for the fully assembled actuator as well.

### 4.4. Dynamic Response of the Actuator

As shown in Figure 10, the rise time for the actuators with smaller CSA was lower than those with larger CSA. The decay time was less than 1 s for all designs. These results can be explained by two factors. First, the restoring force during contraction is higher with larger CSA skeletons, and thus more pressure is required to reach maximum contraction. Second, the airflow may be limited inside the actuator. These results corroborate those previously reported [45].

The spring force of the skeleton enables the actuators to return to their relaxed state passively without requiring manual extension or positive pressure. The response time is mainly attributed to the skeleton as the rise and decay time is due to the compression and extension of the zigzag structure. Rapid actuation times (≈60 ms) have been reported [34] when the actuator is entirely 3D printed with thermoplastic polyurethane. Flexible 3D printed materials are a high-performance alternative for VPAMs.

### 4.5. Case Application in Rehabilitation Exercise

In Figure 12, the range of motion produced by the VPAM from flexion to extension was 43 degrees (119–160°). When the model leg reaches its maximum flexion, the angular velocity decreases, as seen by a decrease in the slope in the angle–time graph. This agrees with the counter-torque provided by the leg’s weight, showing that, in this prone position, the output force of the actuator should be greater than the weight of the lower leg and foot to generate motion. Since gravity in this case helps return the model leg to its original position, only the flexion C6 H10 actuator was used in this experiment.

The output range of motion was slightly below the target value (54° degrees), and represents 80% of it. The VPAM achieved its maximum contraction throughout each exercise, and since the change in length of the actuator is proportional to the output angle in the model leg. It suggests that the actuator produces enough force and that the output range of motion was limited by the actuator’s contraction. This can be addressed by increasing the overall length of the actuator.

A limitation of the approach in prone position is that a rapid return of the leg to its initial position might create a risk of injury for the infant due to the acceleration of the lower leg. In this case, a step response during both contraction and relaxation would be better replaced by a slow ramp to avoid injury, and further work is needed to establish the ideal rates of contraction and relaxation.

On the other hand, the range of motion achieved was approximately 61 degrees (90–151°) in the side-lying position (Figure 13). This output surpassed the target range of motion (54 degrees) by 13 percent, which suggest the VPAM can be a suitable actuator for rehabilitation exercises. In contrast to the output in the prone position, the range is higher, since there is no resistance to the motion due to gravity or the weight of the model leg. The risk of high acceleration remains, and the large velocities and accelerations can be seen in the large slope magnitudes and sharp changes in slope in the transition from initial to final angle in both Figure 12 and Figure 13. This acceleration is halted by the parallel antagonistic actuator, which prevents over-extension or flexion and generates more variability in the transitions of the knee angle. A sinusoidal pressure input wave would be advantageous in that it would not result in abrupt accelerations. Smooth curves are usually observed in native joint flexion or extension and in exosuits for walking assistance [52,53]. These results demonstrate that the desired angle range was achieved in side lying position, but the output angle fell short by 20% in the prone position. A future study may include analysis of the angular velocity to identify the appropriate pressure input for each exercise, such as smooth curves.

There are remaining challenges that will be addressed before advancing along the translational path to clinical use. First, the actuator cannot be used for long-term rehabilitation due to growth of the infant resulting in modified force and length requirements. A closed-loop force feedback would be beneficial for autonomous control of the actuators. If successfully implemented, the system could reduce the workload of the physical therapist for rehabilitation of infants. Soft sensors that accommodate the large strain of the actuator could be implemented to monitor force. Finally, pathological muscle tone (hypo or hypertonicity) in the infant could modify the force requirements for the actuators and necessitate a redesign.

## 5. Conclusions

In this study, we fabricated a low-profile VPAM with a 3D printed skeleton and flexible skin, with dimensions, contraction, and force generation suitable for actuating an infant leg. We characterized the LP-VPAM for rehabilitation exercises in the model leg of a six-month-old infant. The LP-VPAM is suitable for this application since it produces a maximum force of ~26 N at low vacuum pressure magnitudes (<−40 kPa) and can withstand weights ~0.3 kg, which renders it sufficient to move the leg. The actuator configuration was tested in two different positions on a model infant leg—the prone and side-lying position—in order to emulate common positions in which range of motion exercises would be performed on an infant’s knee for myelomeningocele and other lower-limb mobility conditions. The six-cell, 10 mm-height actuator applied to the model leg was able to reach and surpass the target angle range of 54°, with a range of 61° in the side-lying position, showing clinical relevance despite falling short of that target in the prone position due to the additional weight of the leg. The LP-VPAM described in this work produces forces and contraction percentages on a par with existing VPAMs, in addition to its low-profile and low actuator pressure magnitudes. It therefore shows promise for application in rehabilitation exercises in infants.

Future work in this project could include the development of a control scheme, a wearable suit ergonomically tailored for babies, or a portable hardware kit that would allow the platform to be used at home autonomously and safely for the early months of an infant’s life, with a quality of care on a par with the presence of a healthcare professional. Tests of this actuator with a more anatomically true model of an infant leg, with more accurate tissue properties and joint degrees of freedom, would be an important next step towards this greater device development goal.

## Figures and Tables

**Table 1:** Configurations of the geometric parameters.

N° Cells C	Ws (mm)	H (mm)	Ls (mm)	W (mm)
4	30	5	10	38
6	30	5	10	38
4	30	10	10	38
6	30	10	10	38

**Table 2:** Spring constant in N/m for each actuator, from a linear fit of the experimental data.

	Four-Cell	Six-Cell
30xH5	520	380
30xH10	700	340

**Table 3:** Comparison of our LP-VPAM with performance of similar VPAMs in the literature. Scaled force output and percent contraction for the magazine spring, FOAM, and bellows actuators. Scaled force output is calculated by dividing actuator force output by actuation pressure and cross-sectional area, as seen in [41].

Reference	Maximum Force (N)	Scaled Force Output (N/(kPa·mm^2^))	Contraction (%)
Magazine [47]	21	1.02	50
FOAM [46]	145	2.28	50
Bellows [41]	14.9	5.36	71
LP-VPAM	26.4	1.73	61

## Supplementary Materials

The following are available online at https://www.mdpi.com/article/10.3390/mi12080971/s1, Figure S1: Experimental setup for force-contraction profile measurement experiment on mechanical tester. Figure S2: Image of partially-contracted actuator under vacuum, particularly to illustrate the small sections of actuator skin in between the skeleton gaps. Figure S3: Experimental setup for measuring the fixed force output of the VPAMs. Figure S4: Experimental setup for measuring the maximum absolute contraction of the actuators subjected to a load. Figure S5: Experimental setup for the leg model with labels for VPAMs. Figure S6: Tensile testing of FilaFlex. Figure S7: Experimental validation of the FEM. Video S1: Rehabilitation exercise from model leg experiment in the side-lying position.

## References

[1] Okoro C.A., Hollis N., Cyrus A.C., Griffin-Blake S. (2018). Prevalence of Disabilities and Health Care Access by Disability Status and Type Among Adults—United States, 2016. Morb. Mortal. Wkly. Rep. MMWR.

[2] Asbeck A.T., Schmidt K., Walsh C.J. (2015). Soft exosuit for hip assistance. Rob. Auton. Syst..

[3] Asbeck A.T., Dyer R.J., Larusson A.F., Walsh C.J. Biologically-inspired soft exosuit. Proceedings of the 2013 IEEE 13th International Conference on Rehabilitation Robotics (ICORR).

[4] Goher K.M., Fadlallah S.O. (2020). Chapter 11. Assistive Devices for Elderly Mobility and Rehabilitation: Review and Reflection.

[5] Park Y.L., Santos J., Galloway K.G., Goldfield E.C., Wood R.J. A soft wearable robotic device for active knee motions using flat pneumatic artificial muscles. Proceedings of the 2014 IEEE International Conference on Robotics and Automation (ICRA).

[6] Subramanyam K., Rogers E., Kulesza M., Holland D., Gafford J., Goldfield E., Walsh C. (2015). Soft Wearable Orthotic Device for Assisting Kicking Motion in Developmentally Delayed Infants. J. Med. Devices.

[7] Hadders-Algra M. (2018). Early human motor development: From variation to the ability to vary and adapt. Neurosci. Biobehav. Rev..

[8] Domellöf E., Rönnqvist L., Hopkins B. (2007). Functional asymmetries in the stepping response of the human newborn: A kinematic approach. Exp. Brain Res..

[9] Barbu-Roth M., Anderson D.I., Desprès A., Streeter R.J., Cabrol D., Trujillo M., Campos J.J., Provasi J. (2014). Air stepping in response to optic flows that move Toward and Away from the neonate. Dev. Psychobiol..

[10] Goldfield E.C., Park Y.L., Chen B.R., Hsu W.H., Young D., Wehner M., Kelty-Stephen D.G., Stirling L., Weinberg M., Newman D. (2012). Bio-Inspired Design of Soft Robotic Assistive Devices: The Interface of Physics, Biology, and Behavior. Ecol. Psychol..

[11] Apkon S.D., Grady R., Hart S., Lee A., McNalley T., Niswander L., Petersen J., Remley S., Rotenstein D., Shurtleff H. (2014). Advances in the Care of Children with Spina Bifida. Adv. Pediatr..

[12] Sansom J.K., Teulier C., Smith B.A., Moerchen V., Muraszko K., Ulrich B.D. (2013). Muscle activation patterns in infants with myelomeningocele stepping on a treadmill. Pediatr. Phys. Ther..

[13] Instituto Mexicano del Seguro Social Guía De Práctica Clínica (2013). Prevención, Diagnóstico y Tratamiento de la Espina Bífida en Niños. IMSS.

[14] Aizawa C.Y.P., Morales M.P., Lundberg C., Moura M.C.D., Pinto F.C., Voos M.C., Hasue R.H. (2017). Conventional physical therapy and physical therapy based on reflex stimulation showed similar results in children with myelomeningocele. Arq. Neuropsiquiatr..

[15] Michmizos K.P., Rossi S., Castelli E., Cappa P., Krebs H.I. (2015). Robot-Aided Neurorehabilitation: A Pediatric Robot for Ankle Rehabilitation. IEEE Trans. Neural Syst. Rehabil. Eng..

[16] Ren Y., Zhang D. FEXO knee: A rehabilitation device for knee joint combining functional electrical stimulation with a compliant exoskeleton. Proceedings of the 5th IEEE RAS/EMBS International Conference on Biomedical Robotics and Biomechatronics.

[17] Neuhaus P.D., Noorden J.H., Craig T.J., Torres T., Kirschbaum J., Pratt J.E. Design and evaluation of Mina: A robotic orthosis for paraplegics. Proceedings of the 2011 IEEE International Conference on Rehabilitation Robotics.

[18] Mohanta J.K., Mohan S., Deepasundar P., Kiruba-Shankar R. (2018). Development and control of a new sitting-type lower limb rehabilitation robot. Comput. Electr. Eng..

[19] Beyl P., Naudet J., van Ham R., Lefeber D. Mechanical Design of an Active Knee Orthosis for Gait Rehabilitation. Proceedings of the 2007 IEEE 10th International Conference on Rehabilitation Robotics.

[20] Sancho-Pérez J., Pérez M., García E., Sanz-Merodio D., Plaza A., Cestari M. (2016). Mechanical description of ATLAS 2020, a 10-DOF paediatric exoskeleton. Adv. Coop. Robot..

[21] Nickel V.L., Perry J., Garrett A.L. (1963). Development of Useful Function in the Severely Paralyzed Hand. J. Bone Jt. Surg..

[22] Polygerinos P., Wang Z., Galloway K.C., Wood R.J., Walsh C.J. (2015). Soft robotic glove for combined assistance and at-home rehabilitation. Rob. Auton. Syst..

[23] Connolly F., Walsh C.J., Bertoldi K. (2017). Automatic design of fiber-reinforced soft actuators for trajectory matching. Proc. Natl. Acad. Sci. USA.

[24] Li B., Greenspan B., Mascitelli T., Raccuglia M., Denner K., Duda R., Lobo M.A. (2019). Design of the Playskin Air^TM^: A User-Controlled, Soft Pneumatic Exoskeleton. Front. Biomed. Devices.

[25] Kokkoni E., Liu Z., Karydis K. (2020). Development of a Soft Robotic Wearable Device to Assist Infant Reaching. J. Eng. Sci. Med. Diagn. Ther..

[26] Kang J., Martelli D., Vashista V., Martinez-Hernandez I., Kim H., Agrawal S.K. (2017). Robot-driven downward pelvic pull to improve crouch gait in children with cerebral palsy. Sci. Robot..

[27] Lobo M.A., Koshy J., Hall M.L., Erol O., Cao H., Buckley J.M., Galloway J.C., Higginson J. (2016). Playskin Lift: Development and Initial Testing of an Exoskeletal Garment to Assist Upper Extremity Mobility and Function. Phys. Ther..

[28] Rus D., Tolley M.T. (2015). Design, fabrication and control of soft robots. Nature.

[29] Yang D., Verma M.S., So J.H., Mosadegh B., Keplinger C., Lee B., Khashai F., Lossner E., Suo Z., Whitesides G.M. (2016). Buckling Pneumatic Linear Actuators Inspired by Muscle. Adv. Mater. Technol..

[30] Greer J.D., Morimoto T.K., Okamura A.M., Hawkes E.W. Series pneumatic artificial muscles (sPAMs) and application to a soft continuum robot. Proceedings of the 2017 IEEE International Conference on Robotics and Automation (ICRA).

[31] Nguyen P.H., Zhang W. (2020). Design and Computational Modeling of Fabric Soft Pneumatic Actuators for Wearable Assistive Devices. Sci. Rep..

[32] Drotman D., Ishida M., Jadhav S., Tolley M.T. (2019). Application-driven design of soft, 3-d printed, pneumatic actuators with bellows. IEEE/ASME Trans. Mechatron..

[33] Mosadegh B., Polygerinos P., Keplinger C., Wennstedt S., Shepherd R.F., Gupta U., Shim J., Bertoldi K., Walsh C.J., Whitesides G.M. (2014). Pneumatic Networks for Soft Robotics that Actuate Rapidly. Adv. Funct. Mater..

[34] Hu W., Alici G. (2020). Bioinspired Three-Dimensional-Printed Helical Soft Pneumatic Actuators and Their Characterization. Soft Robot..

[35] Tawk C., Spinks G.M., Panhuis M.I.H., Alici G. (2019). 3D Printable Linear Soft Vacuum Actuators: Their Modeling, Performance Quantification and Application in Soft Robotic Systems. IEEE/ASME Trans. Mechatron..

[36] Wang T., Ge L., Gu G. (2018). Programmable design of soft pneu-net actuators with oblique chambers can generate coupled bending and twisting motions. Sens. Actuators A Phys..

[37] Yang H.D., Greczek B.T., Asbeck A.T. (2019). Modeling and Analysis of a High-Displacement Pneumatic Artificial Muscle with Integrated Sensing. Front. Robot. AI.

[38] Belforte G., Eula G., Ivanov A., Visan A.L. (2014). Bellows textile muscle. J. Text. Inst..

[39] Niiyama R., Rus D., Kim S. Pouch Motors: Printable/inflatable soft actuators for robotics. Proceedings of the 2014 IEEE International Conference on Robotics and Automation (ICRA).

[40] Tawk C., Panhuis M.I.H., Spinks G.M., Alici G. (2018). Bioinspired 3d printable soft vacuum actuators for locomotion robots, grippers and artificial muscles. Soft Robot..

[41] Felt W., Robertson M.A., Paik J. Modeling vacuum bellows soft pneumatic actuators with optimal mechanical performance. Proceedings of the 2018 IEEE International Conference on Soft Robotics (RoboSoft).

[42] Yang D., Mosadegh B., Ainla A., Lee B., Khashai F., Suo Z., Bertoldi K., Whitesides G.M. (2015). Buckling of Elastomeric Beams Enables Actuation of Soft Machines. Adv. Mater..

[43] Yang D., Verma M.S., Lossner E., Stothers D., Whitesides G.M. (2017). Negative-Pressure Soft Linear Actuator with a Mechanical Advantage. Adv. Mater. Technol..

[44] Jiao Z., Ji C., Zou J., Yang H., Pan M. (2019). Vacuum-Powered Soft Pneumatic Twisting Actuators to Empower New Capabilities for Soft Robots. Adv. Mater. Technol..

[45] Lee J.-G., Rodrigue H. (2019). Origami-Based Vacuum Pneumatic Artificial Muscles with Large Contraction Ratios. Soft Robot..

[46] Li S., Vogt D.M., Rus D., Wood R.J. (2017). Fluid-driven origami-inspired artificial muscles. Proc. Natl. Acad. Sci. USA.

[47] Kulasekera A.L., Arumathanthri R.B., Chathuranga D.S., Lalitharatne T.D., Gopura R.C. A Low-Profile Vacuum Actuator: Towards a Sit-to-Stand Assist Exosuit. Proceedings of the 2020 3rd IEEE International Conference on Soft Robotics (RoboSoft).

[48] Sargent B., Scholz J., Reimann H., Kubo M., Fetters L. (2015). Development of infant leg coordination: Exploiting passive torques. Infant Behav. Dev..

[49] Hawkes E.W., Christensen D.L., Okamura A.M. Design and implementation of a 300% strain soft artificial muscle. Proceedings of the 2016 IEEE International Conference on Robotics and Automation (ICRA).

[50] Gollob S.D., Park C., Koo B.H.B., Roche E.T. (2021). A Modular Geometrical Framework for Modelling the Force-Contraction Profile of Vacuum-Powered Soft Actuators. Front. Robot. AI.

[51] Sun H., Jensen R. (1994). Body segment growth during infancy. J. Biomech..

[52] Wells J., Hyler-Both D., Danley T., Wallace G. (2002). Biomechanics of growth and development in the healthy human infant: A pilot study. J. Osteopath. Med..

[53] Ding Y., Kim M., Kuindersma S., Walsh C.J. (2018). Human-in-the-loop optimization of hip assistance with a soft exosuit during walking. Sci. Robot..

