# Supplementary material for: A Vacuum-Powered Artificial Muscle Designed for Infant Rehabilitation
Source: Micromachines (Basel). 2021 Aug 16;12(8):971. doi: 10.3390/mi12080971 (PMC8400328; doi:10.3390/mi12080971)
Supplement: Supplementary file 1 [file micromachines-12-00971-s001.zip › micromachines-1285845-supplementary.pdf]

Supplementary Information

A Vacuum-Powered Artificial Muscle Designed for Infant Rehabilitation

Mijaíl Jaén Mendoza, Samuel Dutra Gollob, Diego Lavado, Bon Ho Brandon Koo, Segundo Cruz, Ellen T Roche and Emir A. Vela

Supplemental Methods

Tensile Testing of FilaFlex Samples

To assess the stiffness of the FilaFlex material used in the experiment, we used a tensile tested (Instron 5944). Sections of Filaflex were printed in the same orientation as expected in the skeleton, such that the filament was deposited length-wise. The strips (n = 4) were then clamped in the material tester (Figure S6a) and tensed at a rate of 50mm/min, and the hyper-elastic stress-strain curves were extracted (Figure S6b). Strips were tested to failure. Because the predicted strains for the skeleton were below 0.15, we determined this to fall within the material's linear regime (marked in blue in Figure S6b) and extracted a Young's Modulus of 38.9 MPa (standard deviation of 1.5 MPa), to be used in modelling.

Experimental Validation of FEM

To assess the accuracy of the FEM models, we 3D printed a pair of skeletons following the geometry used for the C6 H10 actuator (C stands for number of cells and H stands for skeleton height) following the procedure described in the Fabrication section. The FilaFlex skeletons were attached at either end to the actuator end fittings, but no skin was attached. Clamping the actuator end pieces in a mechanical tester (Instron 5944), the skeleton was originally placed at a near-neutral position, in slight tension, and then was compressed at a rate of 50 mm/min and the skeleton's restoring force was recorded throughout the contraction. The experiment was repeated twice for each skeleton (n = 2, with a total of four trials).

Figure S7 shows the results of the experiments compared with the model. As can be seen, the restoring forces predicted by the model is lower than that found by the experiment: the experimental skeleton spring constant is 7 N/m while the FEM-predicted value is 3.8 N/m. This discrepancy in the results may be due to anisotropy in the orientation of the printed fibers and larger skeleton thickness than expected due to printer filament, particularly at the rounded corners of the spring.

Despite this discrepancy, the estimated order of magnitude for the restoring force is preserved between FEM and the experiments, supporting the claim that the FEM is sufficient for predicting the scale of the restoring force and assessing a skeleton's validity for not interfering significantly with the actuator's output force.

Actuator Skin Tensile Testing

To measure the skin stiffness needed for the scaling factor, a tensile test was performed, where strips of the PE film used as the skin were cut both axially and transversely to the orientation of the film created by its manufacturing process. The width of each strip was measured, before clamping at either ends with a 50 N load cell on a mechanical tester (Instron 5944). The stress-strain curve for four strips (n = 4) of each orientation was measured and the average elastic deformation stiffness was found. The axial and transverse stiffnesses were 80 and 130 MPa respectively, and together the mean stiffness was found to be 110 MPa. This mean value was used for the scaling factor.

Supplementary Figures

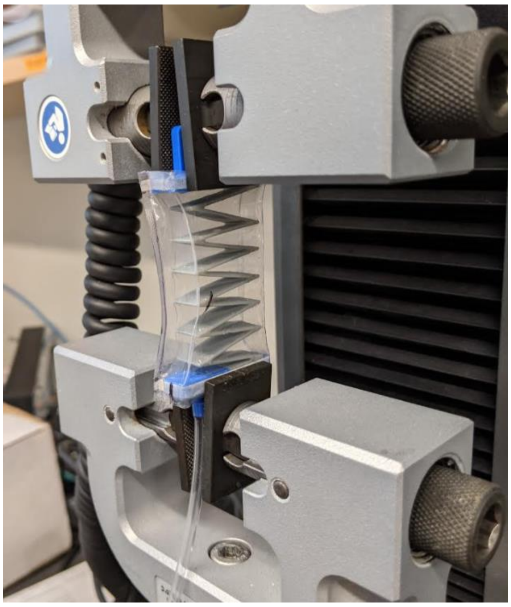

Figure S1. Experimental setup for force-contraction profile measurement experiment on mechanical tester.

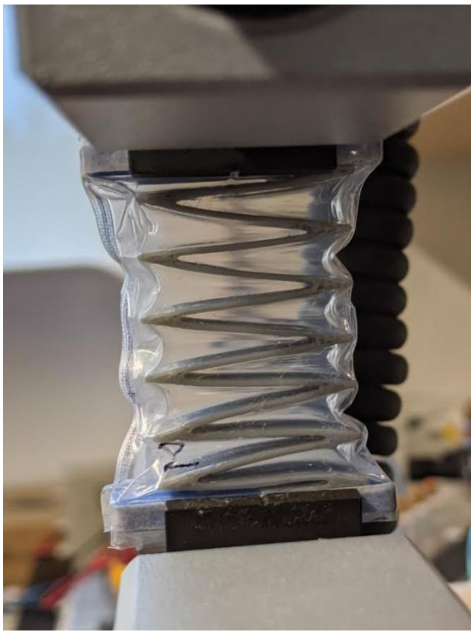

Figure S2. Image of partially-contracted actuator under vacuum, particularly to illustrate the small sections of actuator skin in between the skeleton gaps. These provide increased reaction forces particularly at the end of the contraction, and so are relevant to the reaction force measurement.

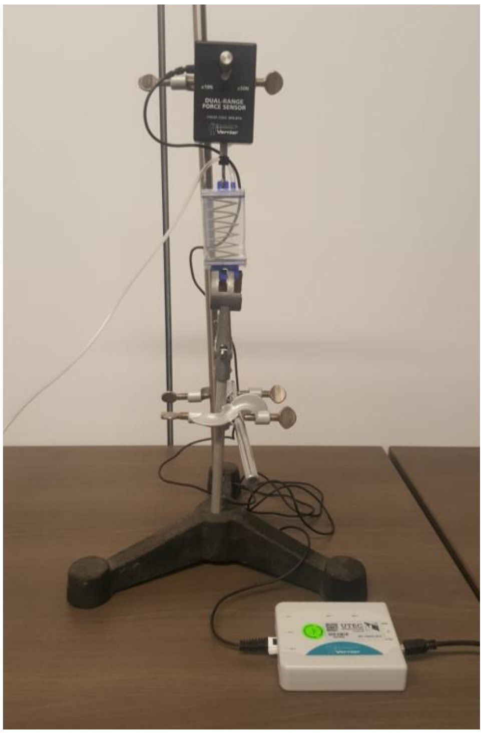

Figure S3. Experimental setup for measuring the fixed force output of the VPAMs. In this case, the length of the actuator is fixed using a metallic tweezers and the force sensor. The actuator is oriented in a vertical position.

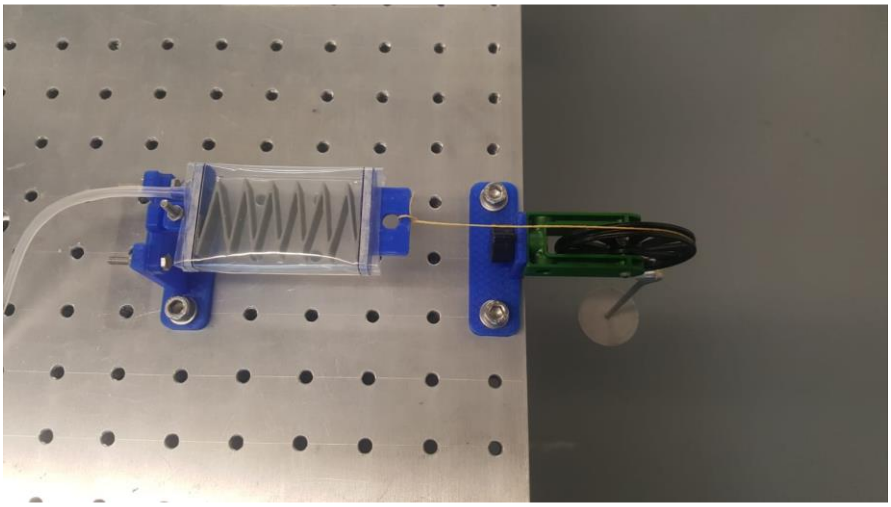

Figure S4. Experimental setup for measuring the maximum absolute contraction of the actuators subjected to a load. The actuator is oriented in a horizontal position.

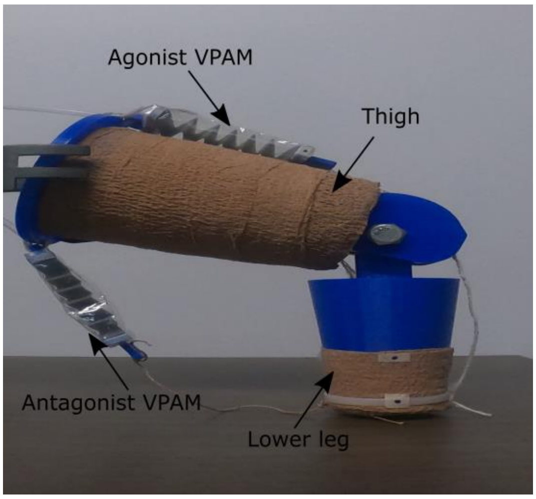

Figure S5. Experimental setup for the leg model with labels for VPAMs. In this case, the leg is oriented as if the patient were in the supine position, while experiments were performed in the prone and sideways positions.

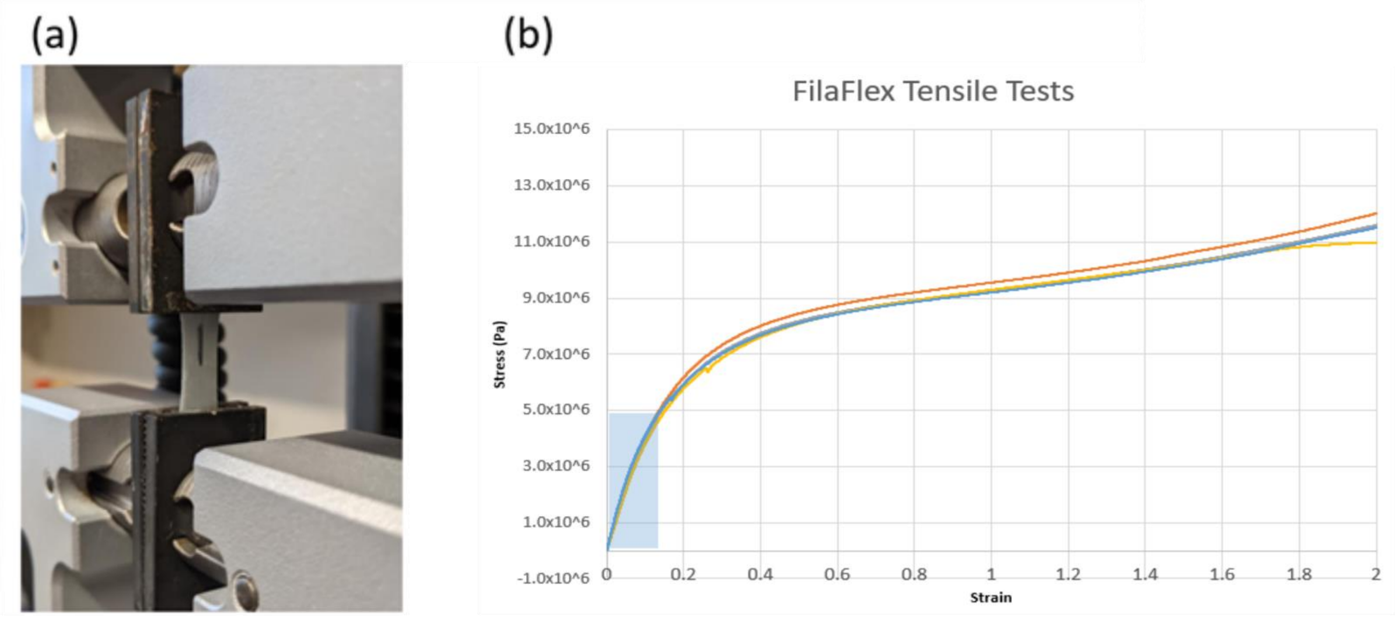

Figure S6. Tensile testing of FilaFlex strips. (a) Image of tensile test setup, (b) Results from tensile tests (n = 4), concatenated to a strain of 2, to allow visualization of linear section, highlighted in blue (below 0.15 strain).

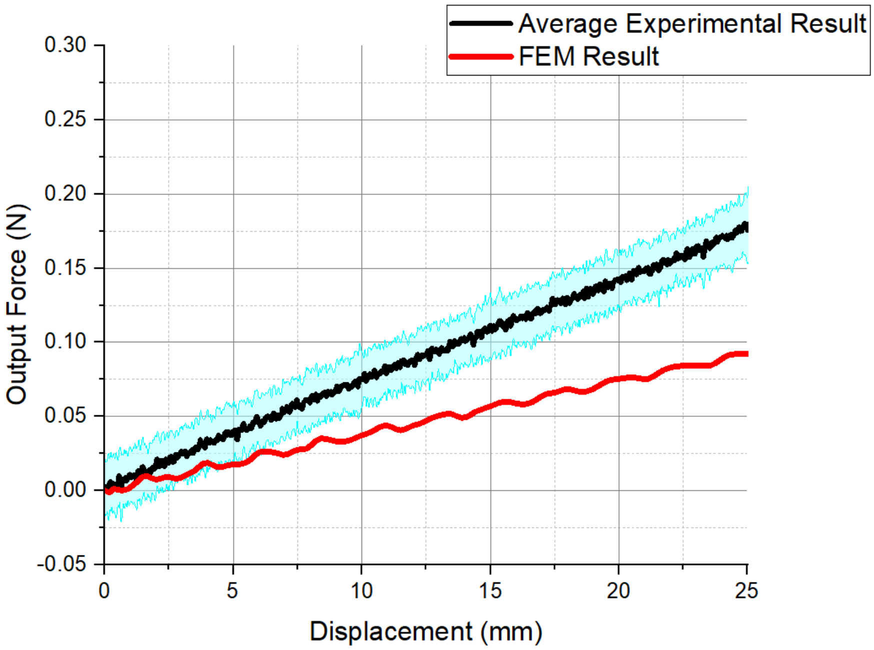

Figure S7. Comparison between experimental and FEM results for compression of zigzag skeletons of the 30 × 10 mm 6-cell configuration. Average line for experiments in black, standard deviation bands in light blue (n = 2, four trials total). Spring constant of experiment is 7 N/m and FEM model is 3.8 N/m.
